# Adiponectin Controls Nutrient Availability in Hypothalamic Astrocytes

**DOI:** 10.3390/ijms22041587

**Published:** 2021-02-04

**Authors:** Nuri Song, Da Yeon Jeong, Thai Hien Tu, Byong Seo Park, Hye Rim Yang, Ye Jin Kim, Jae Kwang Kim, Joon Tae Park, Jung-Yong Yeh, Sunggu Yang, Jae Geun Kim

**Affiliations:** 1 Division of Life Sciences, College of Life Sciences and Bioengineering, Incheon National University, Incheon 406-772, Korea; sannudeul@naver.com (N.S.); dayeon@inu.ac.kr (D.Y.J.); thaihientu@gmail.com (T.H.T.); hr.yang0414@inu.ac.kr (H.R.Y.); 201721047@inu.ac.kr (Y.J.K.); kjkpj@inu.ac.kr (J.K.K.); joontae.park@inu.ac.kr (J.T.P.); yehjy@inu.ac.kr (J.-Y.Y.); 2 Department of Biological Science, University of Ulsan, Ulsan 44610, Korea; bbs0808@naver.com; 3 Department of Nano-Bioengineering, Incheon National University, Incheon 406-772, Korea

**Keywords:** adiponectin, astrocyte, energy metabolism, hypothalamus, glycolysis, metabolic diseases

## Abstract

Adiponectin, an adipose tissue-derived hormone, plays integral roles in lipid and glucose metabolism in peripheral tissues, such as the skeletal muscle, adipose tissue, and liver. Moreover, it has also been shown to have an impact on metabolic processes in the central nervous system. Astrocytes comprise the most abundant cell type in the central nervous system and actively participate in metabolic processes between blood vessels and neurons. However, the ability of adiponectin to control nutrient metabolism in astrocytes has not yet been fully elucidated. In this study, we investigated the effects of adiponectin on multiple metabolic processes in hypothalamic astrocytes. Adiponectin enhanced glucose uptake, glycolytic processes and fatty acid oxidation in cultured primary hypothalamic astrocytes. In line with these findings, we also found that adiponectin treatment effectively enhanced synthesis and release of monocarboxylates. Overall, these data suggested that adiponectin triggers catabolic processes in astrocytes, thereby enhancing nutrient availability in the hypothalamus.

## 1. Introduction

The brain constitutes a metabolically active organ that requires the highest energy demands in the human body. Although the adult brain represents approximately 2% of the total body weight, it consumes approximately a quarter of the total glucose used for its energy supply [1,2,3,4]. Therefore, nutrient availability in the central nervous system (CNS) is directly linked to the maintenance of life. As neurons expend high levels of energy resources, such as glucose and lactate, to initiate and propagate their action potentials [4,5], impairment of the energy supply can lead to perturbation of neuronal excitability. Consistent with these concepts, multiple brain disorders are also deeply associated with abnormalities of energy metabolism in the CNS.

Astrocytes, which comprise the most abundant cell type in the CNS, support normal neuronal functions by regulating the concentration of chemical substances in the synaptic cleft area and providing nutrients between blood vessels and neurons [4,5,6]. Although astrocytes are responsible for the metabolic processing of glucose absorbed by the brain [4,7], they do not require as much energy as they uptake. Rather, the primary driving factor underlying astrocyte participation in glucose uptake and utilization is the provision of energy sources from astrocytes to neurons.

Adiponectin, an adipokine predominantly secreted from adipocytes, has functional roles in regulating glucose and lipid metabolism along with insulin sensitivity. In particular, adiponectin facilitates systemic glucose and lipid homeostasis by regulating several major metabolic organs, such as adipose tissue, liver, and muscle [7,8,9,10,11]. Accordingly, adiponectin elicits beneficial effects in multiple metabolic diseases and their related secondary complications. For example, adiponectin improves hyperglycemia by alleviating glucose intolerance and insulin resistance [9,10]. Furthermore, it mitigates hepatic steatosis and dyslipidemia through regulation of lipid metabolism [10]. Specifically, adiponectin stimulates glucose uptake by skeletal and cardiac muscle and inhibits glucose production by the liver, consequently decreasing blood glucose levels [7,8]. However, although it is well established that adiponectin dynamically participates in the regulation of peripheral energy metabolism, its impact on nutrient metabolism in astrocytes of the hypothalamus, a central unit for the regulation of energy homeostasis, has not yet been clearly elucidated.

We hypothesized that central adiponectin regulates multiple metabolic processes in hypothalamic astrocytes including glucose uptake, glycolytic activity, fatty acid oxidation and metabolites secretion. To verify this hypothesis, in this study we mainly utilized primary astrocytes extracted from the mouse hypothalamus and determined the active roles of circulating adiponectin on hypothalamic astrocytes coupled to whole body energy homeostasis.

## 2. Results

### 2.1. Central Administration of Adiponectin Results in the Activation of Hypothalamic Astrocytes

**Figure 1:**
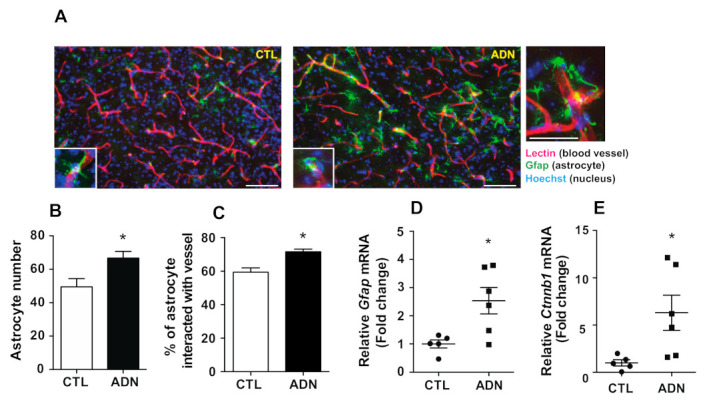
Central administration of adiponectin leads to activation of hypothalamic astrocytes. The whole brain was collected from mice that received an intracerebroventricular (icv) injection of adiponectin (ADN, 3 μg/2 μL) and intracardiac injection of lectin. The distribution of astrocytes was examined by immunohistochemistry using an antibody against glial fibrillary acidic protein (Gfap), a molecular marker for astrocytes. (**A**) Representative images of Gfap immunolabeling in the hypothalamus. Icv administration of adiponectin led to increased (**B**) number of astrocytes and (**C**) contact ratio between astrocytes and blood vessels in the hypothalamus of the mouse brain (*n* = 5 for each group). Icv administration of adiponectin elevated mRNA levels of (**D**) *Gfap* and (**E**) *Ctnnb1* as determined by qRT-PCR (*n* = 5 for CTL; *n* = 6 for ADN). Results are presented as mean ± SEM. * *p* < 0.05. Scale bar = 50 μm.

Based on the evidence that astrocytes respond to metabolic alterations and reactive astrocytes display morphological changes [12], we evaluated the number of astrocytes and their pattern of interaction with blood vessels in the hypothalamus assessed by immunohistochemistry with an antibody against Gfap, a molecular maker for the astrocyte after central administration of adiponectin. Icv administration of recombinant adiponectin into the lateral ventricle of mice resulted in an elevated number of astrocytes in the hypothalamus (Figure 1A,B). We further examined the contact ratio between astrocytes and blood vessels by performing fluorescence immunohistochemistry combined with a visualization of blood vessels by cardiac infusion of lectin to speculate whether adiponectin participates in nutrient shuttling between astrocytes and blood vessels. Notably, icv administration of adiponectin led to an increase in astrocytes interaction with the blood vessel (Figure 1C). In order to further confirm whether adiponectin triggers reactive astrogliosis, we examined the mRNA expression involved in the astrocyte activation. Semi-quantitative RT-PCR results showed an elevation of *Gfap* and *Catenin beta 1*(*Ctnnb1)* transcripts involved in the processes of astrocyte activation (Figure 1D,E), indicating that adiponectin induced reactive astrogliosis.

### 2.2. Adiponectin Enhances Glucose Uptake in Astrocytes

**Figure 2:**
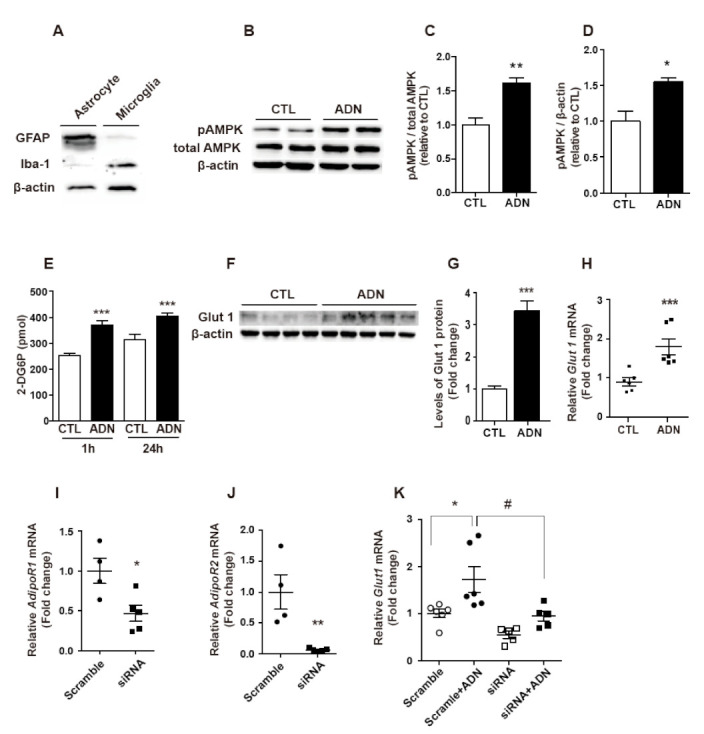
Adiponectin enhances glucose uptake in primary astrocytes. The primary astrocytes and microglia were set up at 5 × 10^5^ cell/well and subjected to western blot analysis by using antibodies for GFAP and Iba-1 proteins. (**A**) GFAP protein was predominantly present in primary astrocytes but Iba-1 protein was almost absent in primary microglia. (**B**–**K**) Primary astrocytes were seeded at 5 × 10^5^ cell/well, starved overnight, and treated with adiponectin (ADN, 1 μg/mL) for 24 h. Cell lysates were subjected to western blot analysis using antibodies against (**B**–**D**) pAMPK, total AMPK, β-actin and (**F**,**G**) glucose transporter-1 (GLUT-1) proteins and qRT-PCR using a primer set of (**H**,**K**) *Glut-1* gene. Exogenous treatment of adiponectin led to increased levels of (**F**,**G**) Glut-1 protein and (**H**) *Glut-1* mRNA in primary astrocytes. (**E**) Adiponectin treatment enhanced glucose uptake in cultured primary astrocytes as determined by 2DG-glucose uptake assay. AdipoR1 and AdipoR2-specific siRNA were transfected into the primary astrocytes to confirm the mRNA expression of *Glut1* induced by adiponectin treatment. qPCR data showed a significant decrease in (**I**) *AdipoR1* and (**J**) *AdipoR2* mRNAs. Elevated (**K**) *Glut1* mRNA induced by adiponectin was effectively rescued by transfection of AdipoR1 and AdipoR2-specific siRNAs. Results are presented as the means ± SEM. *n*= 4 for (**B**–**D**); *n*= 3 for (**E**); *n*= 4–5 for (**F**,**G**); *n*= 6 for (**H**); *n*= 4–5 for (**I**,**J**); *n*= 5–6 for (**K**). All experiments were performed from at least three different preparations of astrocytes. * *p* < 0.05; ** *p* < 0.01; *** *p* < 0.001; ^#^ *p* < 0.05.

To validate the purification of primary astrocytes, we tested the enrichment of GFAP protein in cultured primary astrocytes compared to that in primary microglial cells. GFAP protein was predominantly present in primary astrocytes but not in primary microglia. Additionally, the Iba-1 protein was almost absent in primary astrocytes (Figure 2A). Given the well-known effect of adiponectin on phosphorylation of the AMPK protein, an evolutionarily conserved energy sensor and regulator of energy metabolism, we evaluated the induction of AMPK phosphorylation as determined by immunoblot assay to validate the cellular impact of recombinant adiponectin in primary astrocytes. A treatment of adiponectin resulted in a significant increase in AMPK phosphorylation (Figure 2B–D).

As multiple lines of evidence indicate that adiponectin enhances glucose uptake and utilization in metabolically active peripheral organs, such as muscle and adipose tissue [9], we interrogated whether adiponectin altered glucose uptake in astrocytes using mouse hypothalamic primary astrocytes. We first identified that a significant elevation in glucose uptake was observed in adiponectin-treated astrocytes compared to vehicle-treated astrocytes (Figure 2E). In support of this finding, we observed that exogenous treatment of adiponectin led to increased levels of glucose transporter-1 (Glut-1) protein (Figure 2F,G) and mRNA (Figure 2H), consistent with the effects of adiponectin on glucose metabolism in peripheral organs. To further verify the effect of adiponectin on glucose uptake, we quantified the mRNA expression of *Glut-1* after silencing the expression of adiponectin receptor 1 (AdipoR1) and adiponectin receptor 2 (AdipoR2). We validated the reduced levels of *AdipoR1* and *AdipoR2* mRNAs in response to transfection with siRNAs (Figure 2I,J). An increase of *Glut1* mRNA expression induced by adiponectin treatment was significantly reversed by AdipoR1 and AdipoR2 siRNAs (Figure 2K). These observations suggest that adiponectin has an active role for glucose uptake in hypothalamic astrocytes.

### 2.3. Adiponectin Enhances Glycolytic Activities in Astrocytes

**Figure 3:**
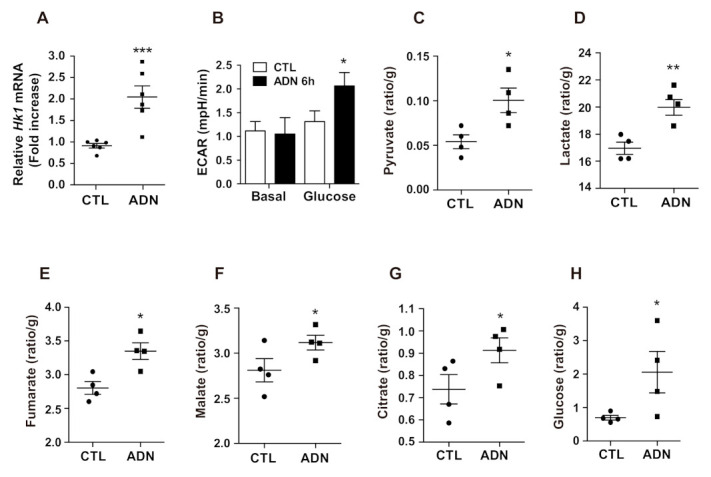
Adiponectin enhances glycolytic activity in primary astrocytes. Primary astrocytes were seeded at 5 × 10^5^ cell/well, starved overnight, and treated with adiponectin (ADN, 1 μg/mL) for 24 h. (**A**) The level of mRNA encoding *hexokinase 1 (Hk1)* was upregulated in adiponectin-treated primary astrocytes compared to that in vehicle-treated primary astrocytes as determined by qRT-PCR (*n* = 6 for each group). (**B**) The extracellular acidification rates (ECAR) were elevated by adiponectin treatment (*n* = 3 for each group). GC-MS results showed increased levels of (**C**) pyruvate, (**D**) lactate, (**E**) fumarate, (**F**) malate, (**G**) citrate, and (**H**) glucose in the hypothalamus of adiponectin-treated mice compared with that of vehicle-treated mice (*n* = 4 for each group). Results are presented as the means ± SEM. qPCR and ECAR experiments were performed from at least three different preparations of astrocytes. * *p* < 0.05; ** *p* < 0.01; *** *p* < 0.001.

We next examined the effect of adiponectin on the glycolytic activities in primary astrocytes. Semi-quantitative RT-PCR results showed that adiponectin treatment elevated the expression of *hexokinase1 (Hk1)*, which catalyzes glucose phosphorylation during glycolysis (Figure 3A). In addition, we further evaluated glycolytic activity utilizing a seahorse XF-24 extracellular flux analyzer. The ECAR in primary astrocytes was significantly elevated by adiponectin treatment (Figure 3B). To verify that enhanced glycolytic activity was triggered by adiponectin, we performed GC-MS to measure the metabolites produced in the glycolytic processes in the hypothalamus of mice injected with adiponectin. Central administration of adiponectin led to an elevation of pyruvate and lactate levels, which correlated with glycolytic activity (Figure 3C,D). Furthermore, icv injection of adiponectin induced increased in levels of fumarate, malate, and citrate generated during the TCA cycle (Figure 3E–G). Notably, elevated glucose levels were observed in the hypothalamus of adiponectin-treated mice (Figure 3H). These data indicated that adiponectin facilitated brain glucose utilization by reinforcing glycolytic activity in astrocytes.

### 2.4. Adiponectin Promotes Synthesis and Release of Monocarboxylates in Primary Astrocytes

**Figure 4:**
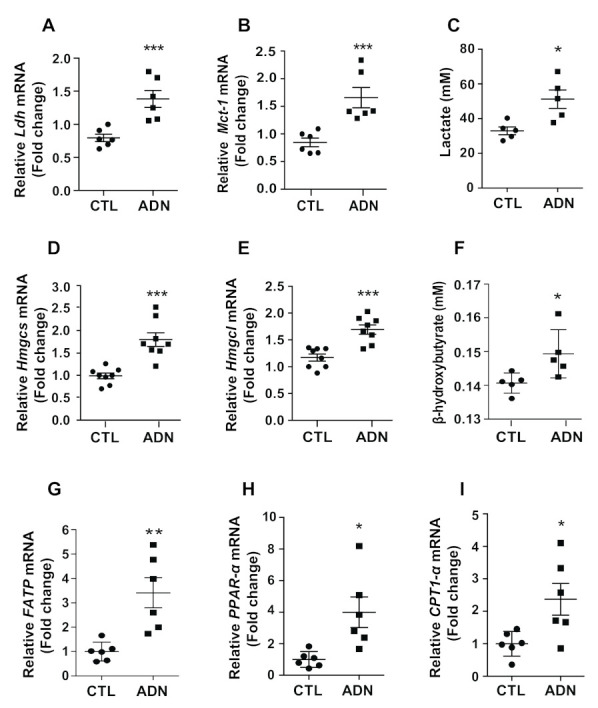
Adiponectin promotes production of lactate and ketone body in primary astrocytes. Primary astrocytes were seeded at 5 × 10^5^ cell/well, starved overnight, and treated with adiponectin (1 μg/mL) for 24 h. The elevated levels of mRNA encoding (**A**) *lactate dehydrogenase (Ldh)* and (**B**) *monocarboxylate transporter-1 (Mct-1)* genes were observed in adiponectin-treated primary astrocyte as determined by qRT-PCR (*n* = 6 for each group). (**C**) The concentration of medium lactate was increased 24 h after adiponectin treatment (*n* = 5 for each group). The elevated mRNA levels of (**D**) *hydroxymethylglutaryl-CoA synthase (Hmgcs)* and (**E**) *hydroxymethylglutaryl-CoA lyase (Hmgcl)* were observed in adiponectin-treated primary astrocytes as determined by qRT-PCR (*n* = 8 for each group). (**F**) The medium concentration of β-hydroxybutyrate was increased 24 h after adiponectin treatment (*n* = 5 for each group). The increased mRNA levels of (**G**) *fatty acid transport protein (FATP)*, (**H**) *peroxisome proliferator-activated receptor-α (PPAR-α)* and (**I**) *carnitine palmitoyltransferase 1-*α (*CTP1-*α) were observed in the adiponectin-treated astrocytes compared with the that in vehicle-treated astrocytes (*n* = 6 for each group). Results are presented as the means ± SEM. All experiments were performed from at least three different preparations of astrocytes. * *p* < 0.05; ** *p* < 0.01; *** *p* < 0.001.

As the production and release of monocarboxylates from astrocytes are coupled to glycolytic processes [4,13], we next interrogated whether adiponectin alters production of monocarboxylates by performing the analysis of mRNA expression in primary astrocytes treated with recombinant adiponectin. We observed that adiponectin treatment led to increased mRNA level of *lactate dehydrogenase (Ldh)*, a catalytic enzyme that reversibly induces conversion of lactate to pyruvate (Figure 4A), and upregulated *monocarboxylate transporter-1 (Mct-1)* (Figure 4B). In support of these molecular observations, an exogenous treatment of recombinant adiponectin led to an elevation of lactate release in cultured primary astrocytes (Figure 4C). In addition, adiponectin-treated astrocytes showed increased mRNA levels of genes involved in the biosynthesis of ketone bodies including *hydroxymethylglutaryl-CoA synthase (Hmgcs)* (Figure 4D) and *hydroxymethylglutaryl-CoA lyase (Hmgcl)* (Figure 4E). In accordance with the patterns of mRNA expression, the medium level of β-hydroxybutyrate was significantly elevated in adiponectin-treated primary astrocytes (Figure 4F). To confirm the increased synthesis and release of ketone bodies, we identified that adiponectin-treated astrocytes showed increased mRNA levels of fatty acid transport protein *(FATP)*, a fatty acid transporter and genes involved in fatty acid oxidation, such as peroxisome proliferator-activated receptor-α *(PPAR-α)* and carnitine palmitoyltransferase 1-α *(CTP1-α)* (Figure 4G–I). These findings indicate that adiponectin promotes synthesis and release of monocarboxylates including lactate and β-hydroxybutyrate in the hypothalamic astrocyte through promoting glucose and lipid utilization.

### 2.5. Central Administration of Adiponectin Leads to an Elevation of Catabolic Processes in the Hypothalamic Astrocytes

**Figure 5:**
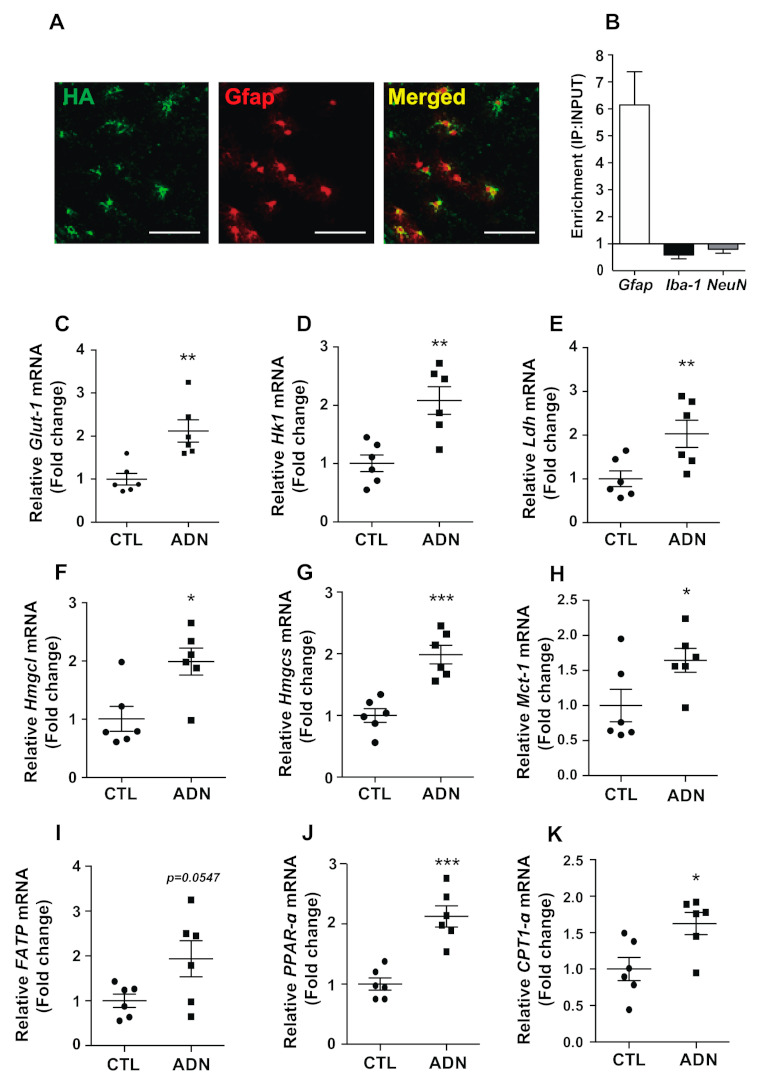
Central administration of adiponectin leads to enhanced glucose utilization and monocarboxylates production in hypothalamic astrocytes of the mice. Ribo-tag analysis were performed to determine alterations in astrocyte-specific mRNA expression in the hypothalamus of *Gfap-CreER^T2^:Rpl22^HA^* mice. (**A**) Representative images revealing co-expression of HA and Gfap immunosignals in the hypothalamus of *Gfap-CreER^T2^:Rpl22^HA^* mice. (**B**) qRT-PCR data showing enrichment of *Gfap* mRNA, a molecular marker for astrocyte (but not *Iba-1*, a molecular maker for microglia and *NeuN*, a molecular marker for neuron) in the purified RNA immunoprecipitated with HA antibody compared with the input RNA extracted from hypothalamus. Elevated mRNA levels of (**C**) *Glut-1,* (**D**) *Hk1,* (**E**) *Ldh,* (**F**) *Hmgcl*, (**G**) *Hmgcs*, (**H**) *Mct-1*, (**I**) *FATP*, (**J**) *PPAR-α* and (**K**) *CTP1-*α were observed in hypothalamic astrocytes from *Gfap-CreER^T2^:Rpl22^HA^* mice that received an icv injection of adiponectin (ADN, 3 μg/2 μL) compared with vehicle-treated control group (CTL). Results are presented as mean ± SEM. *n* = 6 for each group. * *p* < 0.05, ** *p* < 0.01, *** *p* < 0.001. Scale bar = 50 μm.

To further specifically confirmed the impacts of adiponectin on the metabolic process in hypothalamic astrocyte, we used Ribo-Tag technique with *Gfap-Cre;Rpl22^HA^* mice that expressed HA-tagged ribosomal protein Rpl22 in astrocytes. The immunohistochemistry experiment confirmed astrocyte-specific Cre recombination by identifying the immunosignals of HA protein in the Gfap-positive hypothalamic astrocytes (Figure 5A). In addition, we validated the purification of mRNA extracted from the hypothalamic astrocyte by confirming a predominant expression of *Gfap* mRNA, a molecular maker for the astrocytes and minor expression of *Iba-1* mRNA, a molecular marker for microglia and *NeuN* mRNA, a maker for neuron in purified sample compared with the input control sample (Figure 5B). In consistent with mRNA expression data obtained from mouse total hypothalamus and primary astrocytes, we observed that icv administration of adiponectin effectively elevated mRNA levels of *Glut-1* (Figure 5C) and *Hk1* (Figure 5D), indicating enhanced glucose utilization. In addition, Ribo-tag results revealed that central administration of adiponectin resulted in an increase of mRNA levels of genes involved in the synthesis of monocarboxylates including *Ldh* (Figure 5E)*, Hmgcl* (Figure 5F) and *Hmgcs* (Figure 5G), enzymes for the synthesis of lactate and ketone body as well as *Mct-1 gene* (Figure 5H). In support of these observations and in vitro findings, we also identified that central administration adiponectin resulted in elevated levels of mRNAs involved in the fatty acid utilization including *FATP* (Figure 5I)*,* *PPAR-α* (Figure 5J) and *CTP1-*α (Figure 5K). From these data, we successfully confirmed that adiponectin triggers enhanced the catabolic process of nutrients such as glucose and fatty acids, and monocarboxylates production in hypothalamic astrocytes utilizing a mouse model that enabled the purification of astrocyte-specific mRNA.

### 2.6. Adiponectin Rescued 2-DG-Induced Hyperphagia

**Figure 6:**
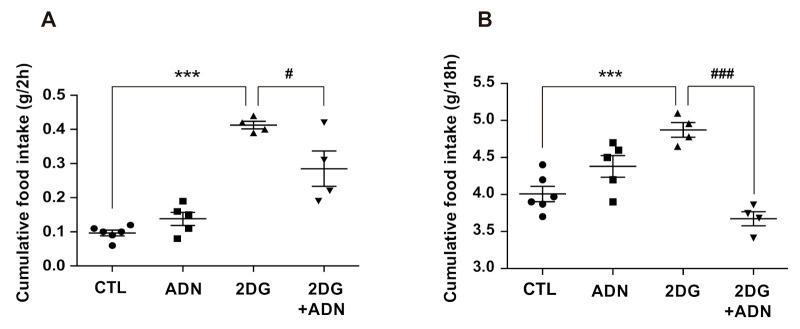
Central administration of adiponectin reversed increased food intake in response to icv injection of 2-DG. Icv injection of adiponectin curbed the increase of (**A**) 2 h and (**B**) 18 h cumulative food intake induced by icv injection of 2-DG. Results are presented as the mean ± SEM. *n* = 4–6 for each group. *** *p* < 0.001 for the 2-DG-treated versus the CTL groups; *^#^ p* < 0.05, ^###^ *p* <0.001 for the 2-DG + ADN-treated versus 2-DG-treated groups.

Central administration of 2-deoxy-D-glucose (2-DG) led to an increase in appetite, suggesting that lower glucose availability drives feeding behavior in association with hypothalamic circuit activity [14]. Thus, we further evaluated the effect of adiponectin on the hyperphagic response induced by 2-DG treatment to identify the physiological relevance of adiponectin-induced alteration in cellular metabolism of hypothalamic astrocytes. In accordance with previous findings, icv administration of 2-DG led to an increase in food intake compared to that in vehicle-treated mice (Figure 6A,B). Increased 2 h and 18 h cumulative food intake induced by 2-DG administration was effectively rescued by adiponectin treatment (Figure 6A,B). These observations suggest that the enhanced catabolic processes of nutrients in hypothalamic astrocytes triggered by adiponectin may be coupled with the homeostatic feeding behavior under the hypoglycemic conditions.

## 3. Discussion

The current study highlights an active role of adiponectin in the regulation of nutrient availability in hypothalamic astrocytes. In turn, this suggests that the alteration of nutrient availability between the hypothalamic neuron-astrocyte-blood vessel axis controlled by adiponectin might be tightly coupled to the function of the hypothalamic circuit that controls whole-body energy metabolism [4,15]. 

Consistent with the concretized evidence that adiponectin affects glucose and lipid utilization in peripheral organs, we here verified the active contributions of adiponectin in glucose and lipid metabolism of hypothalamic astrocytes. We identified that adiponectin facilitates the availability of nutrients in the hypothalamus by promoting the utilization of glucose and fatty acids through the multiple in vitro and in vivo strategies. The hypothalamus constitutes the master organ that controls energy homeostasis by mediating afferent signals derived from metabolically active peripheral organs [16]. In particular, adipose tissue dynamically communicates with the hypothalamus through its own chemical messengers termed adipokines [17,18]. Notably, the circulating levels of most adipokines are proportional to adiposity and long-term elevation of adipokines elicits adverse effects, such as inflammation, oxidative stress, and endoplasmic reticulum stress, which might comprise potential pathogenic elements for the development of metabolic disorders [19].

However, the effects of adiponectin on metabolic controls are distinct from those of general adipokines. In particular, it exerts beneficial effects against multiple cellular stresses and the development of metabolic disorders [15]. In accordance with previous studies, which indicate that adiponectin improves inflammatory responses, [20,21], our results validated the anti-inflammatory properties of adiponectin in hypothalamic astrocytes (Supplementary Figure S1). Moreover, as most adipokines participate in the operation of hypothalamic circuit activity, central adiponectin also acts as an appetite regulator by targeting hypothalamic neurons associated with the brain glucose concentration [22]. Together with our data, these observations suggest that adiponectin-controlled glucose metabolism in hypothalamic astrocytes might be linked to the hypothalamic control of energy homeostasis. Consistent with this, a growing body of evidence has suggested that the nutrient availability between hypothalamic astrocytes and neurons determines the operation of the circuit activity triggering homeostatic feeding behavior [6,23]. Furthermore, recent reports have shown that the metabolic shift induced by fasting or high-fat diet treatment results in alteration of hypothalamic metabolites, such as lactate and ketone bodies [24]. In the present study, we also identified elevated synthesis and release of the monocarboxylates such as lactate and ketone body in primary astrocytes in response to adiponectin treatment. Thus, it is reasonable to hypothesize that adiponectin may affect the excitability of hypothalamic neurons by modulating astrocyte-derived substances, such as gliotransmitters and metabolites. Recently, considerable effort has been paid to unmask the direct contributions of hypothalamic glial cells in regulation of the hypothalamic neuronal circuit [4,25]. However, there is still insufficient information regarding the astrocyte-derived tropic factors that give rise to homeostatic behaviors and responses directly coupled to hypothalamic neuronal functions. In the present study, we proposed that the hypothalamic astrocyte responds to fluctuations in circulating adiponectin and presumably participates in operation of the hypothalamic circuit by modulating the nutrient availability linked to various cellular metabolic processes, including nutrient uptake and release between the extracellular environment and hypothalamic neurons. Notably, a recent study showed that adiponectin evokes the excitation of hypothalamic proopiomelanocortin neurons, which promotes satiety signals under low glucose conditions [22]. In this regard, we identified that central administration of adiponectin effectively curbed hyperphagic behavior induced by 2-DG-mediated limited glucose utilization. As neuron-glia metabolic coupling constitutes a critical cellular event to preserve normal brain functions and the availability of oxygen and energy resources is tightly coupled to neuronal excitability and functions [26], the impairment of nutrient shuttling between astrocytes and neurons may serve as a primary pathological event in the development of multiple neurodegenerative diseases. Thus, our findings raised an open question regarding the potential beneficial effects of central adiponectin in the development of metabolic diseases caused by the degeneration of hypothalamic neurons coupled with the impairment of the neuron-glia metabolic interaction in the hypothalamic circuitry. Therefore, further studies are required to clarify whether disruptions of neuronal functions linked to metabolic disorders can be reversed by adiponectin treatment. Additionally, further investigations are warranted to elucidate whether adiponectin-controlled astrocytes control the activity of hypothalamic neurons linked to energy homeostasis via tropic factors or modulating synaptic inputs. Nevertheless, the current findings collectively suggest that central adiponectin may support normal hypothalamic functions by promoting nutrient availability in hypothalamic astrocytes.

## 4. Materials and Methods

### 4.1. Animals

Eight-week-old male C57BL/6 mice (Dae Han Bio Link, Seoul, Korea) were housed in a 12-h light-dark cycle at 25 °C and 55 ± 5% humidity. The mice were allowed access to normal diet and tap water *ad libitum*. All animal care and experimental procedures were performed in accordance with a protocol approved by the Institutional Animal Care and Use Committee (IACUC) at the Incheon National University (permission number: INU-2016-001).

### 4.2. Cannula Implantation for Intracerebroventricular (Icv) Injection

The mice were anesthetized with an intraperitoneal injection of tribromoethanol (250 mg/kg, Sigma-Aldrich, St. Louis, MO, USA) and placed in a stereotaxic apparatus (Stoelting, Wood Dale, IL, USA). The 2.5-mm cannula (26 gauge) was implanted into the lateral ventricle (X: 1 mm, Y: 0.4 mm to the bregma) and secured to the skull with dental cement. Animals were kept warm until they recovered from the anesthesia and then placed in individual cages. After surgery, a recovery period of seven days was allowed prior to the initiation of experiments.

### 4.3. Icv Injection of Adiponectin

Mice were matched based on body weight and food intake during the adaptation period and divided into adiponectin and phosphate-buffered saline (PBS)-injected groups. Recombinant globular adiponectin (Lugen Sci, Bucheon, Korea) was dissolved in PBS to a concentration of 1.5 mg/mL. Each solution was freshly prepared on the day of administration and free of any contaminants, such as endotoxin. Mice were administered the first icv injection of adiponectin (3 μg/2 μL) 24 h before sacrifice and hypothalamic tissues were harvested 1 h after the second injection of adiponectin (3 μg/2 μL). For the test of feeding behavior, mice were injected with recombinant globular adiponectin (3 μg/2 μL) 1 h before icv injection of 2-deoxy-D-glucose (2.5 mg/2 μL, Sigma-Aldrich, St. Louis, MO, USA), and their food intake was measured.

### 4.4. Immunohistochemistry

Mice were anesthetized, their thoracic cavities were opened, and 30 μg of tomato lectin (Vectorlabs, Burlingame, CA, USA) in 100 μL volume was injected directly into the left ventricle of the heart, over a period of approximately 30 s. The heart continued to beat for approximately 1 min following injection. Subsequently, the animals were transcardially perfused with 0.9% saline (wt/vol), followed by fresh fixative of 4% paraformaldehyde in phosphate buffer (PB, 0.1 M, pH 7.4). Brains were collected and post-fixed overnight before coronal sections (50 μm thickness) were taken by vibratome (5100 mz Campden Instruments, Leicestershire, England). After washing in PB several times, the sections were pre-incubated with 0.3% Triton X-100 (Sigma-Aldrich) in PB for 30 min at room temperature (RT) and incubated overnight with rabbit anti-GFAP antibody (1:1000 dilution, ab7260, abcam, Cambridge, UK) or mouse anti-HA antibody (1:1000; MMS-101R, BioLegend, San Diego, CA, USA) at RT. Immunofluorescence was performed with the secondary antibodies (Alexa Fluor 488-labeled anti-mouse antibody, 1: 500; A11001, Invitrogen, Carlsbad, CA, USA or Alexa Fluor 594-labeled anti-rabbit antibody, 1: 500; A21209, Invitrogen, Carlsbad, CA, USA) for 2 h at RT. The sections were then mounted onto glass slides and covered by coverslips with a drop of mounting medium (Dako North America Inc, Carpinteria, CA, USA). The coverslips were sealed with nail polish to prevent desiccation and movement of the samples under the microscope. The images were recorded using fluorescence microscopy (Axioplan2 Imaging, Carl Zeiss Microimaging Inc., Thornwood, NY, USA) and subjected to analyses.

### 4.5. IHC Image Capture and Analyses

Images were acquired by fluorescence microscopy (Axioplan2 Imaging; Carl Zeiss Microimaging Inc.). For IHC analyses, sections were anatomically matched with the mouse brain using atlas50 (hypothalamic region: between 1.46 and −1.82 mm from bregma). Both sides of the bilateral hypothalamic region were analyzed for two brain sections per mouse. The number of GFAP-positive astrocytes was counted using ImageJ 1.47 v software (National Institutes of Health, Bethesda, MD, USA; http://rsbweb.nih.gov/ij/). Hoechst (Sigma-Aldrich, St. Louis, MO, USA) staining was performed to identify cell nuclei. In sections double-labeled with GFAP antibody and tomato lectin, the number of astrocytes in contact with blood vessels was calculated as a percentage of the GFAP-positive cells in contact with blood vessels per total number of GFAP-positive cells.

### 4.6. Primary Astrocyte Culture

Following decapitation of five C57BL6 mice (5 days old), the hypothalamic tissues were removed, combined in a sterile dish and triturated in Dulbecco’s modified Eagle medium (DMEM) F-12 containing 1% penicillin-streptomycin. The cell suspension was filtered through a 100-μm sterile cell strainer to remove debris and fibrous layers. The suspension was centrifuged and the pellet was resuspended in DMEM F-12 containing 10% fetal bovine serum (FBS) and 1% penicillin-streptomycin. Cells were grown in this culture medium in 75-cm^3^ culture flasks at 37 °C and 5% CO_2_. When cells grew to confluence (about 9 days), the flasks were placed in a 37 °C shaking incubator at 240 rpm for 16 h. The cells were then harvested using 0.05% trypsin-ethylenediamine tetraacetic acid, resuspended in DMEM F-12 containing 10% FBS and 1% penicillin-streptomycin, and centrifuged for 5 min at 1000 rpm. Cells were seeded at a concentration of 5 × 10^5^ cells/mL in culture plates previously treated with poly-L-lysine hydrobromide (50 μg/mL) and grown for 24 h. The medium was changed to DMEM F-12 containing 1% antibiotics without FBS; 24 h later, the same medium plus either 1 µg/mL of recombinant globular adiponectin or vehicle was added. Cells and/or medium were collected 1 or 24 h after treatment. For siRNA transfection, primary astrocytes were seeded (5 × 10^5^ cells/mL) in 6-well culture plates and transfected with siRNAs specific for AdipoR1 (# 72674–1, Bioneer, Daejeon, South Korea) and AdipoR2 (# 68465–1, Bioneer, Daejeon, South Korea) using Lipofectamine 3000 reagent (Invitrogen, Carlsbad, CA, USA) according to the manufacturer’s protocols. A scrambled siRNA (Bioneer) was used as a negative control.

### 4.7. Measurement of Glucose Uptake

Mouse primary astrocytes were seeded at a concentration of 5 × 10^5^ cells/well in 6-well plates. After 24 h treatment of globular adiponectin (1 μg/mL), the cells were processed for a glucose uptake assay following the glucose uptake assay kit instructions (Bio Vision Research Products, Milpitas, CA, USA).

### 4.8. Measurement of Monocarboxylates

The mouse primary astrocytes were seeded at a concentration of 1 × 10^5^ cells/well in 12-well plates and treated with PBS (vehicle control) or globular adiponectin (1 μg/mL) for 24 h. After treatment, the medium were collected and centrifuged to eliminate remaining cells and debris, and processed to measure the concentration of monocarboxylates. For the lactate release assay, the concentration of lactate in the collected medium was examined by using the lactate assay kit (Biomedical Research Service Center, Buffalo, NY, USA) according to the manufacturer’s instructions. The concentration of β-hydroxybutyrate (ketone body) in the collected medium was measured by using β-hydroxybutyrate colorimetric assay kit (Cayman, 700190, MI, USA) according to the manufacturer’s instructions.

### 4.9. Extraction and Analysis of Hydrophilic Metabolites in the Hypothalamus

The extraction and analysis of hydrophilic metabolites in the hypothalamus was performed as previously described [27]. Each sample was extracted with 1 mL of methanol:water:chloroform solution (5:2:2, *v*/*v*/*v*) and 0.03 mL of L-2-chlorophenylalanine in distilled water (0.3 mg/mL) as an internal standard (IS). A mixture of approximately 300 mg glass beads (acid-washed, 425–600 μm, G8772, Sigma-Aldrich, St. Louis, MO, USA) was homogenized for 20 s using a bead beater (Mini Beadbeater-96, BioSpec Products, Bartlesville, OK, USA) and sonicated for 10 min. Thereafter, the samples were incubated in a thermomixer (model 5355, Eppendorf AG, Hamburg, Germany) at 1200 rpm for 30 min at 37 °C and centrifuged at 16,000× *g* at 4 °C for 5 min. The liquid from upper layer (0.8 mL) was transferred into a clean tube and mixed with 0.4 mL distilled water. After centrifugation under the same conditions, 0.9 mL was separated in a new tube and dried using a centrifugal concentrator (VS-802F, Visionbionex, Gyeonggi, Korea) for at least 3 h. The sample was placed in a freeze-dryer (MCFD8512, Ilshin, Gyeonggi-do, Korea) for 16 h to achieve complete concentration. Thereafter, 0.08 mL methoxyamine hydrochloride (20 mg/mL) in pyridine was added for derivatization and incubated at 1200 rpm at 30 °C for 90 min. Next, 0.08 mL *N*-methyl-*N*-(trimethylsilyl) trifluoroacetamide was added and allowed to react at 1200 rpm at 37 °C for 30 min. The derivatized hydrophilic metabolites (1 μL) were analyzed using gas chromatography–mass spectrometry (GC–MS). The GC–MS system consisted of an auto-sampler AOC-20i and GCMS-QP2010 Ultra system (both Shimadzu, Kyoto, Japan) equipped with a DB-5 column (30 m × 0.25 mm id, film thickness 1.0 μm, 122–5033, Agilent, Santa Clara, CA, USA). The flow rate of helium as carrier gas was 1.1 mL/min, and split ratio was 1:10. The injector temperature was 280 °C. The column oven program was as follows: 100 °C for 4 min, increased at a rate of 10 °C/min to 320 °C, and then maintained at 320 °C for 11 min. The ion source temperature was 200 °C, and the interface was set at 280 °C. The scanned mass range was 45–600 *m*/*z*. Lab solutions GCMS solution software (version 4.11; Shimadzu, Kyoto, Japan) was used to identify the hydrophilic metabolites in the hypothalamus. The results were filtered with their retention times and mass spectra with reference to standard compounds and the in-house library. Quantitative analysis was conducted using the ratio of the analyte peak area to the IS peak area.

### 4.10. Ribo Tag Analysis

To specifically evaluated mRNA expression from hypothalamic astrocytes, we utilized the Ribo-Tag translational profiling system [28,29]. In order to generate Ribo-tag mice (*Gfap-CreER^T2^: Rpl22^HA^*), *Rpl22^HA^* mice (Stock No. 011029, Jackson Laboratory) were crossbred with *glial fibrillary acidic protein (Gfap)-CreER^T2^* mice (Stock No.012849), which specifically expresses *Cre* recombinase in astrocytes. Since the *Gfap-CreER^T2^* mice expressed *Cre* recombinase under the control of the tamoxifen inducible GFAP promoter, 8-week-old *Gfap-CreER^T2^: Rpl22^HA^* mice received daily intraperitoneal injections for 5 days of tamoxifen (100 mg/kg,T5648, Sigma-Aldrich, St. Louis, MO, USA) dissolved in corn oil (C8267, Sigma-Aldrich). RNA isolation with the Ribo-Tag system was performed as described by a previous reporters [29,30]. Briefly, the hypothalamus was harvested and homogenized before RNA extraction. RNA was extracted from 10% of the cleared lysate and used as an input control. The remaining lysate was incubated with mouse anti-HA antibody for 4 h at 4 °C followed by the addition of protein G agarose beads (LGP-1018B, Lugen, Gyeonggi-Do, South Korea) and overnight incubation at 4 °C. The beads were washed three times in high-salt solution. The bound ribosomes and RNA were separated from the beads with 30 s of vortexing. Total RNA was extracted using a QIAGEN RNeasy Micro Kit (74034, Qiagen, Hilden, Germany), according to the manufacturer’s instructions and quantified with NanoDrop Lite (Thermo Scientific, Waltham, MA, USA). To evaluate the levels of ribosome-associated mRNA in astrocytes, we synthesized cDNA using a high-capacity cDNA reverse transcription kit (Applied Biosystems, Foster City, CA, USA) and performed quantitative real-time PCR (qRT-PCR).

### 4.11. Quantitative Real-Time Reverse Transcription-Polymerase Chain Reaction (qRT-PCR)

Total RNA was extracted from the hypothalamus or cultured cells according to the Tri-Reagent protocol, and cDNA was then synthesized from total RNA using a high-capacity cDNA reverse transcription kit. Real-time PCR amplification of the cDNA was detected using the SYBR Green Real-time PCR Master Mix (Toyobo Co., Ltd., Osaka, Japan) in a Bio-Rad CFX 96 Real-Time Detection System (Bio-Rad Laboratories, Hercules, CA, USA). The results were analyzed by the CFX Manager software and normalized to the levels of *β-actin* and *L19*, housekeeping genes. The primer sequences used are shown in Table 1. All reactions were performed under the following conditions: initial denaturation at 95 °C for 3 min, followed by 40 cycles of 94 °C for 15 s, 60°C for 20 s, and 72 °C for 40 s.

### 4.12. Immunoblotting

Primary astrocytes were seeded at 5 × 10^5^ cells/well in 6-well plates, allowed to attach overnight, and then incubated in DMEM containing 1 μg/mL adiponectin for 24 h. The cells were washed twice with PBS, followed by scraping and resuspension of the cell pellet in RIPA lysis buffer containing protease inhibitors and centrifugation to remove debris, unbroken cells, and cellular nuclei. Protein content was determined by Bradford’s method using bovine serum albumin as a standard. Samples containing 10 µg of total protein were separated using 12% sodium dodecyl sulfate acrylamide gels. After electrophoresis, proteins were transferred to a nitrocellulose membrane and then blocked with 5% skim milk in TBS (Tris-buffered saline, 0.1% Tween20) buffer for 2 h and incubated in the primary antibodies, including anti-GFAP (1:1000 dilution, Sigma-Aldrich, USA), anti-Iba-1 (1:1000 dilution, Wako, Osaka, Japan), anti-Glut1 (1:500 dilution, Santa Cruz Biotechnology, Dallas, TX, USA), anti-phosphorylated (p)AMPK, anti-total AMPK (1:1000 dilution, Cell Signaling, Danvers, MA, USA), and anti-β-actin (1:10,000 dilution, Sigma-Aldrich, USA).

### 4.13. Measurement of Extracellular Acidification Rates (ECAR)

ECAR measurements were performed using the XF24 Extracellular Flux analyzer (Seahorse Bioscience, North Billerica, MA, USA). Briefly, primary astrocytes were plated into XF24 (V7) polystyrene cell culture plates (Seahorse Bioscience). Primary astrocytes were seeded at 5 × 10^4^ cells/well in poly-L-lysine hydrobromide-coated XF24 plates. The cells were attached overnight and then incubated with globular adiponectin (1 µg/mL) for 6 h in a humidified 37 °C incubator with 5% CO_2_. Prior to performing an assay, growth medium in the wells of an XF cell plate was exchanged with the appropriate assay medium to achieve a minimum 1:1000 dilution of growth medium. Subsequently, 560 µL of the assay medium was added to cells for an XF assay. While sensor cartridges were calibrated, cell plates were incubated in a 37 °C/non-CO_2_ incubator for 60 min prior to the start of an assay. All experiments were performed at 37 °C. Each measurement cycle consisted of a mixing time of 3 min and a data acquisition period of 3 min (12 data points) for the XF24. All compounds were prepared at appropriate concentrations in desired assay medium and adjusted to pH 7.4. For XF24, 80 µL of compound was added to each injection port. In a typical experiment, three baseline measurements were taken prior to the addition of any compound, and three response measurements were taken after the addition of each compound. ECAR were reported as absolute rates mph/min for ECAR.

### 4.14. Statistical Analysis

Data were analyzed using analysis of variance (ANOVA) followed by Student’s *t*-test using Prism GraphPad. *p* value ≤ 0.05 was considered statistically significant. The values are represented as the means ± standard error of the mean (SEM).

## Figures and Tables

**Table 1:** Real-time PCR primer sequences.

Target Gene	Direction of Primer	Sequence (5′→3′)
*Gfap*	Forward	TCA ATG ACC GCT TTG CTA GC
	Reverse	ACT CGT GCA GCC TTA CAC AG
*Iba-1*	Forward	TCT GCC GTC CAA ACT TGA AG
	Reverse	TCT AGG TGG GTC TTG GGA AC
*NeuN*	Forward	ATG GTG CTG AGA TTT ATG GAG G
	Reverse	CGA TGG TGT GAT GGT AAG GAT C
*Ctnnb1*	Forward	ATC CAA AGA GTA GCT GCA GG
	Reverse	TCA TCC TGG CGA TAT CCA AG
*Glut-1*	Forward	CTT CAT TGT GGG CAT GTG CTT C
	Reverse	AGG TTC GGC CTT TGG TCT CAG
*Hk1*	Forward	AGA GGC CTA GAC CAC CTG AAT GTA A
	Reverse	ACT GTT TGG TGC ATG ATT CTG GAG
*Ldh*	Forward	AGC CCT GAC TGC ACC ATC ATC
	Reverse	CGG AAT CGA GCA GAA TCC AGA
*Mct-1*	Forward	AAT GAT CGC TGG TGG TTG TC
	Reverse	TTG AAA GCA AGC CCA AGA CC
*Hmgcs*	Forward	TTT GAT GCA GCT GTT TGA GG
	Reverse	CCA CCT GTA GGT CTG GCA TT
*Hmgcl*	Forward	CCA GCT TTG TTT CTC CCA AG
	Reverse	TCA GAC ACA GCA CCG AAG AC
*L19*	Forward	GGT GAC CTG GAT GAG AAG GA
	Reverse	TTC AGC TTG TGG ATG TGC TC
*FATP*	Forward	GCA GCA TTG CCA ACA TGG AC
	Reverse	GTG TCC TCA TTG ACC TTG ACC AGA
*PPAR-α*	Forward	ACG CTC CCG ACC CAT CTT TAG
	Reverse	TCC ATA AAT CGG CAC CAG GAA
*CPT1- α*	Forward	CCA GGC TAC AGT GGG ACA TT
	Reverse	GAA CTT GCC CAT GTC CTT GT
*AdipoR1*	Forward	TGA CTG GCT GAA AGA CAA CG
	Reverse	TTG GTC TCA GCA TCG TCA AG
*AdipoR2*	Forward	ATC CCT CAC GAT GTG CTA CC
	Reverse	TAA AAG ATC CCC AGG CAC AG
*IL-1β*	Forward	ATA CTG CCT GCC TGA AGC TCT TGT
	Reverse	AGG GCT GCT TCC AAA CCT TTG AC
*IL-6*	Forward	TGG TCT TCT GGA GTA CCA TAG C
	Reverse	TCT GAA GGA CTC TGG CTT TGT C
*β-actin*	Forward	GAT CTG GCA CCA CAC CTT CT
	Reverse	GGG GTG TTG AAG GTC TCA AA

## Data Availability

All data reported in the manuscript and in the Supplementary materials.

## Supplementary Materials

The following are available online at https://www.mdpi.com/1422-0067/22/4/1587/s1, Figure S1: Adiponectin leads to decreased mRNA levels of proinflammatory cytokines in primary astrocytes.
