# Supplementary material for: Adiponectin Controls Nutrient Availability in Hypothalamic Astrocytes
Source: Int J Mol Sci. 2021 Feb 4;22(4):1587. doi: 10.3390/ijms22041587 (PMC7915184; doi:10.3390/ijms22041587)
Supplement: Supplementary file 1 [file ijms-22-01587-s001.pdf]

## Supplementary Figure 1

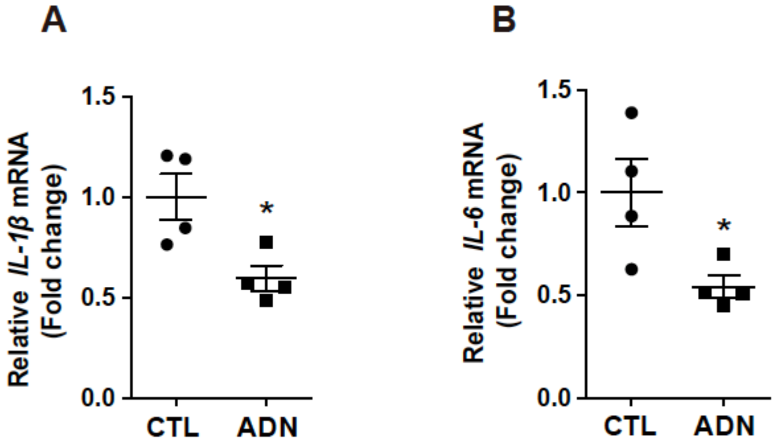


**Supplementary Figure 1. Adiponectin leads to decreased mRNA levels of proinflammatory cytokines in primary astrocytes.** Primary astrocytes were seeded at 5×10$^{5}$ cell/well, starved overnight, and treated with adiponectin (1μg/ml) for 24 h. The reduced levels of mRNA encoding (A) *interleukin-1β (IL-1β)* and (B) *interleukin-6 (IL-6)* genes were observed in adiponectin-treated primary astrocyte as determined by qRT-PCR. Results are presented as the means ± SEM, $^{*}$*P<0.05*.
